# Effectiveness of *Scolopendrid* Pharmacopuncture for Neuropathic Dysfunction: Clinical Evidence and Potential Mechanism

**DOI:** 10.3390/toxins17020083

**Published:** 2025-02-12

**Authors:** Jung-Hyun Kim, Tae-Yoon Kim, Bonhyuk Goo, Sang-Soo Nam

**Affiliations:** 1 Department of Acupuncture and Moxibustion Medicine, Kyung Hee University Hospital at Gangdong, Seoul 05278, Republic of Korea; dan_mi725@naver.com (J.-H.K.); goobh99@naver.com (B.G.); 2 Department of Korean Traditional Medicine Practice, Jaseng Medical Foundation, Seoul 06110, Republic of Korea; pacifier9@naver.com; 3 Department of Acupuncture and Moxibustion Medicine, College of Korean Medicine, Kyung Hee University Hospital at Gangdong, Kyung Hee University, Seoul 05278, Republic of Korea

**Keywords:** *Scolopendrid* venom, animal venom, pharmacopuncture, neuropathic dysfunction, traditional Korean medicine

## Abstract

Animal venoms, particularly *Scolopendrid* venom, have gained significant attention as therapeutic agents in complementary and alternative medicine, especially for applications in pain management and neuroprotection. In traditional Korean medicine, *Scolopendrid* venom is administered through pharmacopuncture, a method that combines injection therapy with principals of acupuncture. The present review focuses on the multifaceted effects of *Scolopendrid* pharmacopuncture, derived from *Scolopendra polymorpha*, on the peripheral nervous system, and its potential role in addressing the neuropathic dysfunction that often arises from peripheral nerve injuries. *Scolopendrid* venom exhibits various pharmacological properties, including analgesic, anti-inflammatory, and neuroprotective effects. Experimental studies have shown that *Scolopendrid* pharmacopuncture significantly reduces neuropathic pain in animal models by modulating ion channels and inflammatory pathways. Clinical investigations have further revealed its efficacy in alleviating pain associated with conditions such as Bell’s palsy and carpal tunnel syndrome. Despite its promising therapeutic potential, the lack of comprehensive clinical research on the toxicity and safety profiles of SPP remains a critical limitation. Future studies should focus on evaluating the safety of *Scolopendrid* venom as a standalone treatment and incorporate broader data sources to enhance our understanding of its implications in clinical practice.

## 1. Introduction

Recently, the exploration of animal venoms as sources of therapeutic agents has gained significant attention in complementary and alternative medicine, particularly for their potential application in pain management and neuroprotection [1]. In traditional Korean medicine, various animal venom-derived pharmacopuncture injections are used to normalize physiological functions and improve pathological conditions in humans [2]. Pharmacopuncture injection therapy involves a procedure in which specific medicinal solutions are injected into trigger points and blood vessels, identified through the stimulation of meridian points or superficial tissues, using a syringe. Therefore, this method allows the combined effects of acupuncture and pharmacological treatment [3]. Among animal venom-derived pharmacopuncture injections, *Scolopendrid* pharmacopuncture (SPP) involves the extraction of active components from *Scolopendrid* venom, which are then injected into specific meridian and primary trigger points that are effective for certain diseases from a traditional medicinal perspective.

Scolopendrids, belonging to the suborder *Scolopendromorpha* (class *Chilopoda*, phylum *Arthropoda*), are predatory centipedes characterized by elongated, segmented bodies with one pair of legs per segment [4]. Within *Scolopendromorpha*, the family *Scolopendridae* encompasses diverse genera, including *Scolopendra*, *Cormocephalus*, and *Digitipes*, exhibiting distinct morphological and genetic traits [5]. Integrating morphological and molecular phylogenetic data, particularly from mitochondrial and nuclear genes, has refined our understanding of scolopendrid taxonomy and evolutionary relationships, revealing cryptic diversity within this group [6].

*Scolopendrid* venom exhibits a complex composition comprising diverse proteins, peptides, and enzymes with varied biological functions [7]. Studies have identified numerous toxin-like molecules within this venom, with significant interspecies variation in composition, including 246 unique proteins found in *Scolopendra mojiangica* [8]. Notable toxin types include alpha-like scorpion neurotoxins and metalloproteinases, exhibiting neurotoxic and enzymatic activities, respectively, with some peptides, such as SsmTP from *Scolopendra subspinipes mutilans*, demonstrating concentration-dependent cytotoxic and growth factor-like effects [9]. These diverse components confer potential pharmacological applications, including anticoagulant, analgesic, and ion channel-modulating properties [10]. *Scolopendrid* venom, specifically derived from *Scolopendra polymorpha*, is a notable candidate because of its unique biochemical composition and multifaceted effects on the peripheral nervous system (PNS) [11].

An impairment of the PNS can lead to neuropathic dysfunction. Peripheral nerve injury or damage is a primary mechanisms linking the PNS to neuropathic dysfunction [12]. This can occur because of various factors, including trauma, infections, toxins, and autoimmune diseases. When the peripheral nerves are damaged, their ability to conduct signals is compromised, leading to aberrant pain processing [13]. In facial nerve palsy, carpal tunnel syndrome, and radial nerve palsy, this can manifest as neuropathic pain, often described as burning, shooting, or tingling sensations that do not correspond to any identifiable injury [14].

The PNS plays a significant role in neuroinflammation, which can exacerbate neuropathic dysfunction. Inflammatory processes can lead to further injury of peripheral nerves, creating a cycle of pain and dysfunction [15]. Conditions such as diabetic neuropathy illustrate how prolonged hyperglycemia can induce inflammatory responses that damage peripheral nerve fibers, contributing to chronic pain and sensory deficits [16].

The PNS is also involved in modulating pain pathways [17]. Abnormalities in the peripheral nociceptors functioning, specialized nerve endings that detect harmful stimuli, can result in heightened sensitivity to pain, known as allodynia and hyperalgesia [18]. Altered pain perception is a hallmark of neuropathic dysfunction and signifies a maladaptive response to injury.

Neuropathic dysfunction, characterized by abnormal pain processing and resistance to traditional analgesic therapies, presents a significant clinical challenge [14,19]. Understanding the mechanisms through which *Scolopendrid* venom modulates pain pathways and neuroinflammatory responses is crucial for developing innovative treatment strategies.

The multifaceted effects of *Scolopendrid* venom on the PNS requires comprehensive investigation. By contributing to the understanding of the mechanisms by which this venom influences pain pathways, inflammation, and nerve health, this review provides valuable insights into its therapeutic potential. These findings may pave the way for novel treatment modalities that address the challenges associated with neuropathic pain and nerve injury, ultimately enhancing clinical outcomes in affected patients.

## 2. Mechanisms of Action of Scolopendrid Venom in the Peripheral Nerve System

*Scolopendrid* venom comprises a complex mixture of bioactive peptides and proteins that exhibit extensive pharmacological properties. It contains various bioactive peptides and proteins that target and modulate ion channels and play essential roles in neuronal excitability and neurotransmission [20].

### 2.1. Influence on Ion Channel Gating in Terms of Peripheral Nerve System

*Scolopendrid* venom exerts analgesic effects through various mechanisms that target ion channels in the PNS [21]. The components of the venom, particularly neurotoxins, interact with voltage-gated calcium channels, resulting in reduced nociceptive transmission. Notably, *Scolopendrid* venom contains peptides that act as high-voltage-activated calcium channel blockers, specifically targeting N-type calcium channels [22]. This action reduces calcium influx, thereby decreasing neurotransmitter release and pain signaling. In addition, the recombinant form of Phα1β, known as CTK 01512-2, has potential in modulating TRPA1 channels, further contributing to its analgesic properties [23].

Furthermore, venom-derived peptides selectively inhibit transient receptor potential vanilloid 1 (TRPV1), a receptor crucial for pain perception. By binding to the outer pore region of TRPV1, these peptides modulate its activity, offering a promising pathway for the development of analgesic drugs [24]. Peptides from *Scolopendrid* venom may also target acid-sensing ion channels (ASICs), which play a significant role in pain pathways; these toxins can alter ASIC gating, leading to analgesic effects in both central and peripheral nerve systems [25]. Additionally, venom components may inhibit voltage-gated sodium channels, particularly target voltage-gated sodium (NaV) 1.7, which is essential for pain signaling. This inhibition effectively reduces pain transmission in peripheral neurons [26].

### 2.2. Immunomodulatory and Neuroprotective Effects of Scolopendrid Venom on PNS

A prominent mechanism of action involves the modulation of inflammatory responses. Scolopendrid venom and its extracts have been shown to suppress the production of key inflammatory mediators, including tumor necrosis factor alpha (TNF-α), interleukin 6 (IL-6), nitric oxide (NO), and prostaglandin E2 (PGE2). This suppression is mediated, at least in part, by the inhibition of the nuclear factor kappa beta (NF-κB) signaling pathway, a crucial regulator of inflammatory processes [27,28]. This pathway’s inhibition consequently reduces the expression of pro-inflammatory cytokines, further dampening the inflammatory cascade. This attenuation of glial activation contributes to the overall reduction in neuroinflammation within the PNS.

Beyond its anti-inflammatory actions, *Scolopendrid* venom exhibits direct neuroprotective effects. It enhances cell viability, likely through the activation of nerve growth factor (NGF) signaling pathways, which are essential for neuronal survival, growth, and repair [29]. This activation of NGF signaling pathways contributes to the observed functional recovery in nerve injury models treated with *Scolopendrid* venom [30]. Additionally, components of the venom may mitigate oxidative stress, a significant factor contributing to neurodegeneration [31]. This mitigation is potentially achieved by bolstering antioxidant defenses and reducing mitochondrial damage [32,33]. These combined effects—immunomodulation and direct neuroprotection—underscore the therapeutic potential of *Scolopendrid* venom in addressing neuropathic conditions affecting the PNS.

## 3. Evidence from Experimental Studies

Experimental studies have indicated that *Scolopendrid* venom exhibits favorable effects on neuropathic dysfunction, demonstrating its potential as a therapeutic agent for managing neuropathic pain and promoting recovery following nerve injuries [34]. In a rat model of neuropathic pain, *Scolopendrid* pharmacopuncture significantly reduced the withdrawal response associated with both mechanical and chemical allodynia, as shown by decreased c-fos expression in the midbrain and a reduced white blood cell count [35].

Moreover, the scolopendrid water–alcohol extract (SWAE) applied at the BL23 acupuncture point markedly increased the mechanical threshold of the foot in neuropathic rats for at least 4 h, indicating a partial alleviation of pain. This analgesic effect was comparable to that of conventional anticonvulsant medications such as gabapentin. The analgesic properties of the SWAE are linked to the suppression of NO production and the expression of inducible and neuronal nitric oxide synthases (NOSs), further supporting its role in modulating pain pathways [36].

In addition, pharmacopuncture with scolopendrid extracts improved functional recovery and reduced pain severity in sciatic nerve injury models. Treatment was associated with decreased expressions of inflammatory markers, including cyclooxygenase-2 (COX-2) and TNF-α, and enhanced neurofilament expression, which is indicative of neuronal health. Furthermore, *Scolopendrid* extracts demonstrated anti-inflammatory effects by inhibiting the NF-κB signaling pathway and reducing the production of inflammatory mediators, both in vitro and in vivo [37].

Collectively, these results highlight the therapeutic potential of *Scolopendrid* venom in alleviating neuropathic pain and facilitating recovery from peripheral nerve injuries and shows its multifaceted mechanisms of action involving anti-inflammatory, analgesic, and neuroprotective effects. The study designs and results are presented in Table 1.

## 4. Evidence from Clinical Studies

In a clinical investigation focused on the efficacy of SPP in alleviating postauricular pain, patients diagnosed with Bell’s palsy exhibited significant reductions in pain when treated with SPP in conjunction with conventional therapies. The treated group reported a marked decrease in postauricular pain compared to those receiving conventional treatment alone and experienced a shorter duration of pain, indicating enhanced therapeutic benefits [46].

A comparative study on the effects of scolopendrid and sweet bee venom pharmacopuncture on carpal tunnel syndrome revealed that both treatment modalities led to significant improvements in patient-reported outcomes, as measured using the visual analog scale and pain rating scale. Although no significant differences were noted between the two groups, the results suggest that SPP effectively alleviates the symptoms associated with neuropathic conditions [47].

Moreover, SPP has shown promise for reducing mechanical allodynia and thermal hyperalgesia in animal models of neuropathic pain. In these studies, scolopendrid extracts improved pain thresholds and showed anti-inflammatory effects by downregulating the expression of pro-inflammatory cytokines and enhancing neuroprotective mechanisms [48]. Collectively, these findings underscore the potential of scolopendrid venom as a therapeutic agent for managing neuropathic pain and promoting recovery in various clinical settings. Treatment properties and outcomes are listed in Table 2.

## 5. Discussion

### 5.1. For Safe Injections

While existing studies highlight the complex composition of scolopendrid venom, comprising up to 246 types of peptides and proteins with diverse bioactivities, the subsequent discussion of preclinical and limited clinical data suggests a relatively low toxicity profile for the crude venom, particularly when administered via pharmacopuncture at controlled dosages. This apparent discrepancy between the venom’s complex composition and its observed low toxicity warrants further consideration. Several factors may contribute to this phenomenon. First, the specific peptides and proteins responsible for pain and local swelling may be present in relatively low concentrations within the crude venom. Second, the pharmacopuncture administration technique, involving small injection volumes and potentially targeting specific sites, may limit systemic exposure to the venom’s components. Third, it is possible that synergistic interactions between the various components of the venom, while contributing to its therapeutic effects, may also modulate its overall toxicity, perhaps through counteracting or buffering mechanisms. Research has indicated that SPP does not exhibit significant toxicological effects when administered at controlled doses, suggesting a favorable safety profile for this practice. A study involving SD rats demonstrated that SPP administered at doses of 0.21, 0.42, and 0.84 mg/kg did not result in any deaths or significant adverse effects, with parameters such as weight, food intake, and organ health remaining stable. Notably, the lethal dose (LD50) for SPP was determined to be >0.84 mg/kg, indicating a relatively high safety margin [49].

Although direct human studies on SPP are limited, insights have been gained from research on other venom-based treatments, such as bee venom pharmacopuncture, which reported a 10% incidence of mild side effects, all of which resolved without lasting effects [50]. However, the potential for allergic reactions or adverse effects cannot be overlooked, as has been observed in other venom studies. Therefore, although current animal studies suggest that SPP is safe, further clinical research is essential to fully elucidate its implications in humans.

In terms of delivery, the injectable route ensures rapid absorption and immediate therapeutic effects, which are essential in emergencies. Injectable forms facilitate the quick systemic distribution of venom, which is crucial in cases of envenomation where time is essential. Furthermore, intramuscular or subcutaneous injections can directly target affected tissues, thereby enhancing the therapeutic effect of the venom [51].

From a safety perspective, studies have indicated that injectable *Scolopendrid* venom administration does not exhibit significant toxicological changes, suggesting a safer profile than oral delivery, which may lead to gastrointestinal complications. In addition, injectables allow precise dosing and minimize the risk of overdose or adverse reactions that can occur with oral administration [3].

### 5.2. Limitations and Future Research Directions

The absence of clinical research data regarding the toxicity of SPP during actual application poses a significant challenge in substantiating its beneficial effects on neuropathic dysfunction. Current studies are limited by small sample sizes and have primarily assessed the safety of combined treatments rather than evaluating the safety of SPP as a standalone intervention. This limitation arises from the nature of traditional Korean medical practices, which typically use multiple interventions simultaneously, instead of relying solely on a single treatment modality. Therefore, future safety studies should focus on rigorously assessing the safety of SPP when administered independently instead of within the context of complex treatment regimens.

Furthermore, the current understanding of the safety of the subcutaneous administration of this venom is limited by the scarcity of clinical studies. The available LD50 data, while informative regarding acute toxicity in preclinical models, does not adequately address potential long-term or subacute effects in humans. Comparing the toxicity of this venom to that of bee venom, a substance with a distinct pharmacological profile and commonly used in Korean traditional medicine, is of limited value. The observed lack of significant toxicity in animal models, as evidenced by stable physiological parameters and high LD50 values, may not fully reflect the potential for localized reactions or long-term effects. Further research, including detailed analysis of individual venom components and their interactions, as well as more extensive clinical trials evaluating both local and systemic effects of SPP, is necessary to fully reconcile the complex nature of the venom with its observed safety profile.

Finally, preclinical and clinical studies highlighted in this research underscore a significant limitation, owing to the lack of findings from internationally recognized databases. This reliance on medical databases from the Republic of Korea and China raises concerns about selection bias and the potential inability of this study to provide contrasting or complementary perspectives within a broader international context. Acknowledging these limitations is crucial as it emphasizes the need for caution when generalizing the findings of studies.

## 6. Conclusions

The findings presented in this review highlight the therapeutic potential of *Scolopendrid* venom, particularly through its pharmacopuncture application in the management of neuropathic dysfunction. Its multifaceted mechanisms of action, including analgesic, anti-inflammatory, and neuroprotective effects, underscore its potential as a novel treatment modality for conditions characterized by neuropathic pain and peripheral nerve injuries. Experimental and clinical studies have demonstrated that *Scolopendrid* venom alleviates pain and facilitates functional recovery, indicating its capacity to address significant clinical challenges associated with neuropathic conditions. The favorable safety profile observed in animal studies further supports the viability of SPP as a therapeutic option.

While the findings presented here offer promising insights into the therapeutic potential of *Scolopendrid* venom, particularly for neuropathic pain management, further research employing a comprehensive approach with diverse, internationally recognized databases is warranted to strengthen these observations. Such investigations, focusing on rigorous clinical trials, including assessments of its safety as a standalone treatment, will be essential to definitively establish the efficacy and safety profile of *Scolopendrid* venom for integration into clinical practice and ultimately optimize patient care.

## Figures and Tables

**Table 1:** Designs of preclinical experimental studies.

	Animal Model	Dosage	Experimental Design	Outcomes and Conclusions
Kim (2004) [35]	Sympathetic-independent pain-induced rat model of neuropathy [38] (male Sprague Dawley [20] rat)	0.5, 0.1, and 0.05 mL/kg	A model of neuropathic pain was produced by resecting the tibial and sural nerves under isoflurane anesthesia. Allodynia was assessed through medial malleolus stimulation. *Scolopendrid* aqua-acupuncture was performed at Hwando (GB30) once daily for 1 week, followed by an evaluation of the withdrawal response and examination of c-fos expression and WBC.	(1) *Scolopendrid* aqua-acupuncture at GB30 significantly reduced withdrawal responses to both mechanical and chemical allodynia in the treatment groups compared to the control group. (2) Significant differences were found in c-fos expression between the treatment and control groups, as well as between the sham and treatment groups. (3) Treatment significantly affected white blood cell count, indicating its potential efficacy in modulating pain responses and inflammatory markers. (4) SPP may be an effective intervention for alleviating neuropathic pain and influencing related biological responses.
Lee (2004) [36]	Segmental spinal nerve ligation induced-rat model of neuropathy [39] (male SD rats)	Diluted to a concentration of 10 mg/mL in normal saline solution and 100 μL injected at the acupoint. This corresponds to the same amount as administering 4 mg/kg of *Scolopendrid* venom to experimental animals weighing 250 g	Based on the clinical application of scolopendrid, the effects of SWAE applied to acupuncture points were evaluated in a rat model of neuropathic pain. Neuropathic pain was induced by the tight ligation of the L5 spinal nerve. Once the rats exhibited pain behaviors, 100 microliters of SWAE at a concentration of 10 mg/mL was administered to the ipsilateral BL23 point under enflurane anesthesia. Foot withdrawal latency of the hind limb was measured as an indicator of pain levels following each treatment.	(1) The injection of SWAE significantly increased the mechanical threshold of the foot in the rat model of neuropathic pain for 4 h, indicating a partial alleviation of pain. The analgesic effect was found to be dose-dependent and comparable to that of gabapentin, with the ipsilateral BL23 point showing the most significant improvement in mechanical sensitivity compared to other points. (2) SWAE exerts a potent analgesic effect in the neuropathic pain model and that this analgesia is mediated by the modulation of endogenous NO through the suppression of inducible NO synthase (iNOS) and neuronal NO synthase (nNOS) protein expression.
Lee (2013) [37]	Sciatic nerve injury-induced rat model of neuropathy [40] (male SD rats)	10 μL/day for 7 d	This study investigated *Scolopendra subspinipes* pharmacopuncture on functional recovery, pain severity, and the expression of neurofilament, COX-2, and TNF-α following sciatic nerve crush injury in rats. The methodologies included walking track analysis, plantar testing, Western blotting for COX-2 and TNF-α, and immunohistochemistry for neurofilaments.	*S. subspinipes* pharmacopuncture effectively ameliorate symptoms of sciatic nerve crush injury by improving the sciatic functional index, reducing pain severity, and modulating the expression of COX-2, TNF-α, and neurofilament, with both treatments exhibiting similar therapeutic efficacy.
Li (2015) [41]	Right L5 spinal nerve ligation induced-rat model of neuropathy [39] (male SD rats)	On KI1 acupoint, 200 μL extract (1 mg) was injected with 29-gauge needle	The effects of *Scolopendrid* pharmacopuncture on the onset and maintenance stages of neuropathic pain were assessed using behavioral assessments after a single treatment and five once-daily treatments, respectively. Western blotting and immunohistochemistry testing were performed.	Repeated *S. Subspinipes* pharmacopuncture treatments effectively alleviated mechanical allodynia and thermal hyperalgesia, primarily through the deactivation of microglia and astroglia, as indicated by reduced glial fibrillary acidic protein and ionized calcium binding adaptor protein expression.
Hwang (2018) [42]	Sciatic nerve injury-induced rat model of neuropathy [43] (male SD rats)	0.1, 1, and 10 g/kg	This study assessed the anti-inflammatory effects of *S. subspinipes* muricatus by inducing inflammation in RAW 264.7 cells with lipopolysaccharide and evaluating inflammatory factors using enzyme-linked immunosorbent assay (ELISA) and Western blotting, as well as by examining behavioral outcomes and inflammatory factor expression in a sciatic nerve crush injury model in rats.	The results demonstrate that *S. subspinipes muricatus* (SSM) treatment exhibits significant anti-inflammatory effects by suppressing PGE2 and NO production, inhibiting NF-κB activation, and reducing the expression of inflammatory markers such as iNOS, TNF-α, IL-6, and COX-2, while enhancing functional recovery in behavioral tests, indicating its potential as a therapeutic candidate for neuropathic pain.
Seo (2019) [44]	Male C57BL/6 mice (8 weeks of age, weighing 20–25 g) with trimethyltin-induced hippocampal neurodegeneration and seizures [45]	Administered SWE (50 mg/kg) for 1 week	This study investigated how SWE affected TMT-induced cytotoxicity in primary cultures of mouse hippocampus neurons (7 d in vitro) and how SWE affected hippocampus degeneration in adult C57BL/6 mice treated with TMT (2.6 mg/kg, intraperitoneal).	(1) SWE pretreatment significantly reduced TMT-induced cytotoxicity in cultured hippocampal neurons in a dose-dependent manner and attenuated hippocampal cell degeneration and seizures in adult mice, as evidenced by reduced lactate dehydrogenase levels and improved behavioral outcomes. (2) SWE pretreatment led to a significant reduction in Iba-1- and GFAP-positive cell bodies in the dentate gyrus, indicating a protective effect against microglial and astrocyte activation, suggesting the potential pharmacotherapeutic use of SWE in addressing hippocampal degeneration and dysfunction.

**Table 2:** Designs of clinical studies included in this review.

Author (Year)	Design	Interventions	Frequency of Treatment and Duration	Patients	Outcome Measure	Results
Seo (2005) [46]	Case report	Administered at a depth of 0.5–1.0 mm at specified acupuncture points, with a total volume of 0.2 mL delivered in increments of 0.05–0.1 mL	Treatments were conducted twice or thrice weekly at 2–3 d intervals.	An 18-year-old male patient with an inability to extend the left wrist joint.	Range of motion (ROM) and muscle strength of the wrist and finger joints, as well as the presence of muscle atrophy.	After 4 weeks of sessions, the ROM and the power of the wrist recovered to a nearly normal range.
Ku (2010) [47]	Randomized controlled trial	Using a 1 mL, 30-gauge syringe, insert the needle at a 45° angle between PC7, which is located between the flexor carpi radialis tendon and the palmaris longus tendon, until reaching the midpoint of the transverse carpal ligament, and then inject 0.2 mL of *Scolopendrid* venom	Treatments were administered twice weekly for 4 weeks, eight sessions in total.	(1) Aged 20–70 years. (2) Without symptoms in the neck, shoulders, upper arms, forearms, or elbows, but experiencing pain, tingling, or increased nighttime pain in the hands, with a visual analog scale score of ≥5. (3) With at least one positive result for Tinel’s or Phalen’s signs. (4) Diagnosed with carpal tunnel syndrome based on nerve conduction velocity study.	(1) Visual analog scale, (2) PRS ^1^, (3) Tinel’s sign, (4) Phalen’s sign, (5) Nerve conduction velocity study ^2^	SPP led to significant improvements in the visual analog scale and pain rating scale, but no significant differences were observed between the two groups. Only the control group showed a notable reduction in positive responses for Tinel’s and Phalen’s signs, whereas neither group showed improvement in nerve conduction velocity. Overall, the results suggest that SPP may alleviate symptoms of carpal tunnel syndrome.
Kwak (2012) [48]	Prospective open-label clinical trial	Using a 30-gauge, 1 mL syringe, 0.4–0.8 mL was administered around the affected TE 17 acupoint	Three sessions were conducted every other day in the afternoon, with the first session on the day of admission, the second session on the 3rd day of admission, and the third pharmacopuncture treatment on the 5th d of admission.	Among patients diagnosed with Bell’s palsy who received inpatient treatment, 32 individuals diagnosed with peripheral facial nerve paralysis and experiencing postauricular pain within 1 week of onset were selected. These patients were divided into two groups: 16 patients who received only conventional traditional Korean treatments, including acupuncture and herbal medicine, and 16 patients who received the same conventional treatments in addition to SPP at the TE17 acupuncture point. The treatment outcomes were then compared between the two groups.	(1) NRS, (2) Yanagihara’s unweighted grading system	(1) The SPP group demonstrated a significant reduction in postauricular pain and a shorter duration of such pain compared to the conventional treatment group (group A), highlighting its potential effectiveness in managing this symptom. (2) The differences were not statistically significant; however, the findings suggest that SPP may provide substantial benefits for alleviating postauricular pain associated with Bell’s palsy.

^1^ Pain was measured based on criteria such as pain intensity, daily frequency, pain patterns related to movement, and duration. The pain index was calculated using the following formula: pain intensity × (time + frequency + movement). ^2^ During the motor nerve conduction study of the median nerve, an active recording electrode was placed on the belly of the abductor pollicis brevis, whereas the reference electrode was positioned over the tendon of this muscle. The sensory nerve conduction study used an orthodromic measurement technique that involved stimulating the nerve in the fingers and recording it at the wrist. The latency and amplitude of the motor nerve and the amplitude and conduction velocity of the sensory nerve were measured to compare differences between the two groups.
